# Validation of a hybrid approach to standardize immunophenotyping analysis in large population studies: The Health and Retirement Study

**DOI:** 10.1038/s41598-020-65016-x

**Published:** 2020-05-29

**Authors:** DeVon Hunter-Schlichting, John Lane, Benjamin Cole, Zachary Flaten, Helene Barcelo, Ramya Ramasubramanian, Erin Cassidy, Jessica Faul, Eileen Crimmins, Nathan Pankratz, Bharat Thyagarajan

**Affiliations:** 1 Division of Epidemiology and Community Health, University of Minnesota, Minneapolis, MN USA; 2 Divison of Computational Pathology, Department of Laboratory Medicine and Pathology, Minneapolis, MN USA; 3 Institute for Social Research, Survey Research Center, University of Michigan, Ann Arbor, MI USA; 4 Davis School of Gerontology, University of Southern California Davis, Los Angeles, CA USA; 5 Division of Molecular Pathology and Genomics, Department of Laboratory Medicine and Pathology, Minneapolis, MN USA

**Keywords:** Bioinformatics, Bioinformatics, Bioinformatics, High-throughput screening, High-throughput screening

## Abstract

Traditional manual gating strategies are often time-intensive, place a high burden on the analyzer, and are susceptible to bias between analyzers. Several automated gating methods have shown to exceed performance of manual gating for a limited number of cell subsets. However, many of the automated algorithms still require significant manual interventions or have yet to demonstrate their utility in large datasets. Therefore, we developed an approach that utilizes a previously published automated algorithm (OpenCyto framework) with a manually created hierarchically cell gating template implemented, along with a custom developed visualization software (FlowAnnotator) to rapidly and efficiently analyze immunophenotyping data in large population studies. This approach allows pre-defining populations that can be analyzed solely by automated analysis and incorporating manual refinement for smaller downstream populations. We validated this method with traditional manual gating strategies for 24 subsets of T cells, B cells, NK cells, monocytes and dendritic cells in 931 participants from the Health and Retirement Study (HRS). Our results show a high degree of correlation (r ≥ 0.80) for 18 (78%) of the 24 cell subsets. For the remaining subsets, the correlation was low (<0.80) primarily because of the low numbers of events recorded in these subsets. The mean difference in the absolute counts between the hybrid method and manual gating strategy of these cell subsets showed results that were very similar to the traditional manual gating method. We describe a practical method for standardization of immunophenotyping methods in large scale population studies that provides a rapid, accurate and reproducible alternative to labor intensive manual gating strategies.

## Introduction

Flow cytometry (FCM) provides a high dimensional quantitative measure for single cell analysis of the immune system. Manual gating using analyzer-defined boundaries or “gates” to identify cell populations of interest is commonly analyzed using proprietary software (e.g. FlowJo Tree Star Inc version 10). In the context of large-scale epidemiological investigations involving thousands of samples, this time consuming and labor-intensive process depends on the skill of the analyst, thereby introducing subjectivity that can increase variability among analysts and limit reproducibility of flow cytometry assays.

Recent technological advancements in computational methods help reduce the subjectivity intrinsic to manual gating for multi-dimensional flow cytometry data and promote standardization of immunophenotyping analysis in large population studies. Several automated analyses tools such as OpenCyto^1^ and FLOCK^2,3^ and hybrid tools such as DAFi^4^ and FlowGM^5^ have been validated in recent years with good concordance when compared to manually gated datasets. Additional tools such as CytoML^6^ allow data to be shared across platforms, which makes a streamlined analysis using both automated and manual analysis possible. Recently, the Flow Cytometry Critical Assessment of Population Identification Methods (FlowCAP) study evaluated 36 different computational approaches for automated analysis of flow cytometry. OpenCyto and flowDensity were two of the top performing gating algorithms in the FlowCAP study^3,7–9^. Since the FlowCAP validation, flowDensity^10^ has been integrated and supported as a plug-in to the OpenCyto^1^ framework, supporting the reproducible end-to-end flow data analysis pipeline. Though older versions of OpenCyto have been validated across multiple cell populations on a limited number of samples, OpenCyto has been upgraded to incorporate multiple new Bioconductor packages and newer versions of OpenCyto have not undergone a full end-to-end validation. Limitations of validation of all these automated methods performed to date include studies with limited sample size in populations (n < 250), software packages that require substantial post-processing manual intervention that limits the utility of the automated analysis^2,4,11–13^, and software packages that are better suited for discovery-oriented data analysis rather than facilitating rapid analysis of known cell subsets in a large number of study participants^2,14,15^. Furthermore, since different markers and fluorochromes are used to identify cell subsets in different studies, the ability of automated software to reliably detect rare cell subsets may differ across studies and thus require validation under the conditions used in an individual study^3,9,15,16^. Lastly, previous validations of software for automated analysis have highlighted the limitation of automated analysis in accurately identifying and quantifying rare cell subsets and necessitating manual analysis of rare cell subsets.

**Figure 1:**
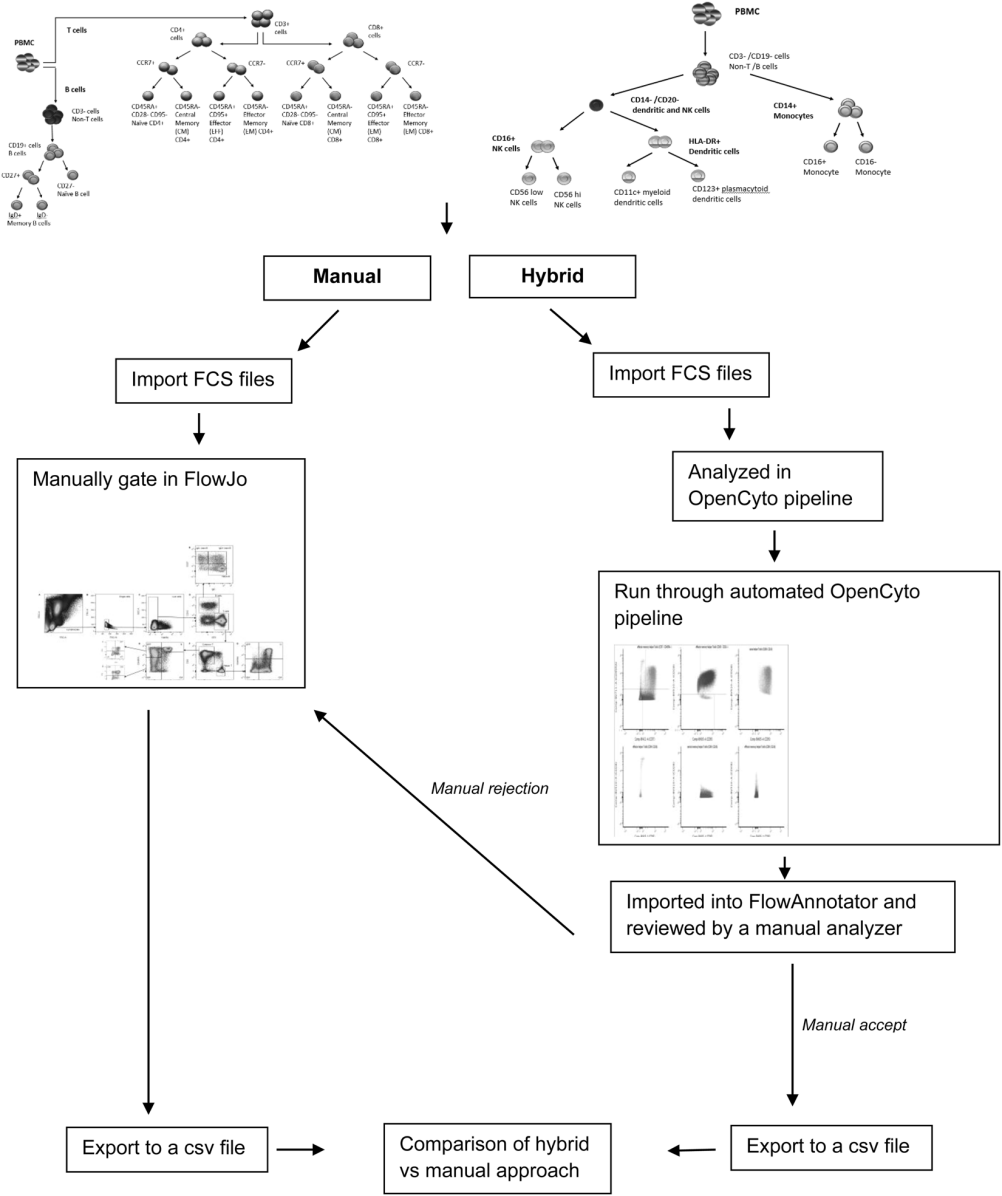
Workflow comparison between analysis platforms, FlowJo (manual gold standard), and the hybrid approach of automated gating using OpenCyto and manual refinement using FlowAnnotator. End to end visualization of the stepwise process beginning with importing of the raw cytometry data (FCS) and export of a text-based csv file.

Hence, we conducted a validation study to evaluate the feasibility of using a hybrid approach that incorporates hierarchical gating templates implemented in OpenCyto along with custom developed visualization software, FlowAnnotator, to analyze flow cytometry data in 10,000 participants from the Health and Retirement Study (HRS). We addressed limitations of previous validations by (a) validating the latest version of OpenCyto (ver. 1.12.1) against manual gating for 24 immune cell subsets of interest on 931 study participants and (b) developing a visualization and annotation software (FlowAnnotator) that allows users to quickly screen cell populations identified by OpenCyto to identify individual samples and cell populations for manual intervention or further refinement of parameters to improve the performance of OpenCyto (Fig. 1).

## Results

**Figure 2:**
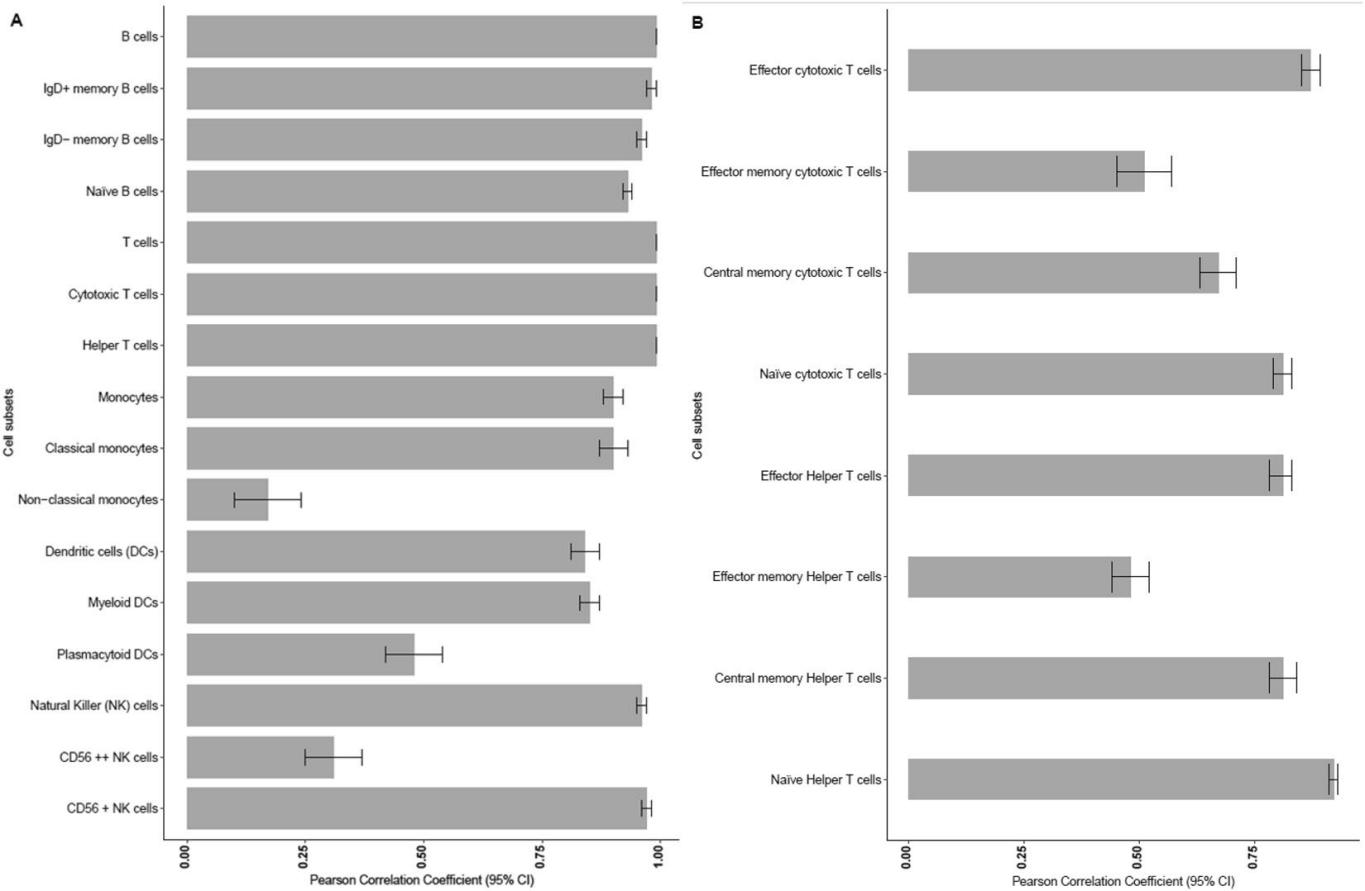
(**A**) Pearson correlation comparison between the hybrid and manual approaches across 16 main immune cell subsets. Error bars represent the 95% confidence interval. (**B**) Pearson correlation comparison between the hybrid and manual method across the T cell subsets. Error bars represent the 95% confidence interval.

**Figure 3:**
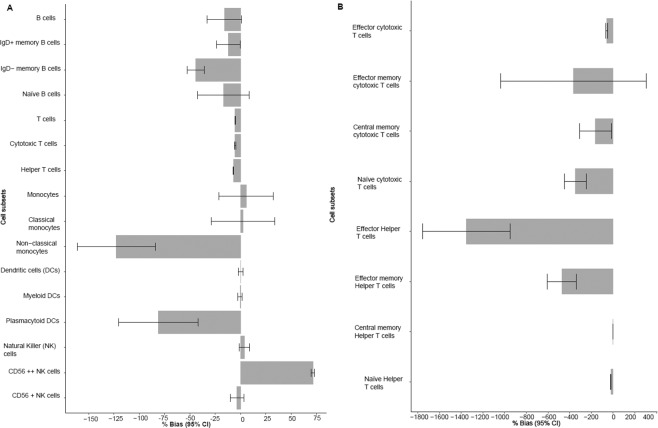
(**A**) Comparison of percent bias in hybrid vs. manual approaches for the main 16 immune cell subsets. Error bars represent the 95% confidence interval. (**B**) Comparison of percent bias in hybrid vs. manual approaches for the cytotoxic and helper T cell subsets estimated using the k-means approach. Error bars represent the 95% confidence interval.

Eleven percent of all FCS files in the validation dataset were tagged for manual at least one immune cell population. There were seven cell populations used for the validation of the hierarchical gating templates implemented in OpenCyto. Validation cell populations included B cells, T-cells, and the two major T-cell subsets; helper CD4+ T cells and cytotoxic CD8+ T cells, natural killer (NK) cells, monocytes and dendritic cells. All seven validation populations had between 0.01–5.45% FCS files tagged for manual analysis. The subsets directly downstream of these populations had 3.7–10.35% FCS files tagged for manual analysis. Overall, there was a high correlation between the hierarchical gating templates implemented in OpenCyto and manual analysis (FlowJo) for all seven validation populations (Fig. 2A) (correlation ≥0.90 for all major populations except dendritic cells (r = 0.84)). All seven major cell populations also showed minimal bias when comparing results between manual and hybrid methods (bias ranging from −15.79% to 0.02% for all seven cell subsets) (Fig. 3A) and the absolute difference between the hybrid and manual approaches was also minimal (Supplementary Table 1). Since the naïve, central memory, effector memory and effector subsets of cytotoxic CD8+ and helper CD4+ T-cell populations were better defined by three markers (CCR7, CD45RA and CD28) these cell subsets could not be adequately gated using two-dimensional gating strategies. Hence these cell populations were validated using a k-means clustering approach that simultaneously utilized all three markers to accurately identify these T-cell subsets. The Pearson correlations for the four common subsets of cytotoxic and helper T-cells (CCR7/CD45RA) that underwent k-means clustering (naïve cytotoxic, effector cytotoxic, naïve helper, effector helper, central memory helper) were >0.80 (Fig. 2B). The rarer subsets such as cytotoxic central memory, cytotoxic effector memory and helper effector memory T-cells had lower Pearson correlations (r = 0.48–0.67) (Fig. 2B). Though there was a large negative percent bias in estimating several cytotoxic and helper T-cell subsets (Fig. 3B) the absolute difference between the two methods for these rarer subsets was minimal (3.16–6.34 × 10^9^ cells/L) (Supplementary Table 1).

B cell subsets (IgD−, IgD+, Naïve), NK cell subset (CD56LO), classical monocytes, and myeloid dendritic cells had Pearson correlations >0.85 (Fig. 2A). The percent bias for these subsets was considerably higher when compared to the seven major validation populations (−81.25% − 2.55%) (Fig. 3A). Immune cell populations with lower correlations were non-classical monocytes (r = 0.17), plasmacytoid dendritic cells (r = 0.48), and NK cell subset (CD56HI) (r = 0.31) (Fig. 2A). While these cell subsets showed a relatively high percent bias when estimated using the hybrid approach as compared to manual analyses (Fig. 3A), each of these populations displayed minimal absolute difference (0.0004 × 10^9^ cells/L − 0.0002 × 10^9^ cells/L) between the hybrid and manual methods (Supplementary Table 1). Cell subsets with lower absolute cell counts had higher percent biases, indicating that these biases maybe driven by the low values (Fig. 3A,B and Supplementary Table 1). Removing outliers significantly improved the correlation between hybrid and manual gating methods. For example, the correlation for plasmacytoid dendritic cells increased to 0.80 from 0.48 (after 98 FCS files were removed) indicating that this low correlation was influenced by these outliers (Supplementary Table 1). Overall the absolute difference observed between the 24 populations was minimal, demonstrating that the hierarchical gating templates implemented in OpenCyto and FlowJo were similar. The F-measures generated comparable results to what was observed by the Pearson correlation coefficients (Supplementary Table 1).

### Independent validation of the hybrid approach in an independent dataset

Since the initial set of 931 samples was used to optimize the parameters in the hybrid approach, we validated the final parameters chosen for the hybrid approach in an independent dataset of 100 samples in which we compared the correlation between the hybrid and manual approaches. Supplementary Table 2 shows that there was a high correlation between the hybrid and manual approaches for the seven major cell populations (r > 0.80) like the results observed in the dataset used to optimize the hybrid approach. All other cell subsets also showed similar correlations as observed in the initial dataset of 931 samples with high correlation seen for most cell subsets except for rare cell subsets such as non-classical monocytes and central memory cytotoxic and helper T cells subsets (Supplementary Table 2).

### Comparison of Inter- and intra- technician variability by both hybrid and manual approaches

Among the 169 measurements done on eight PBMC controls analyzed by five technicians at multiple timepoints using both the manual and hybrid approaches, the manual approach showed low intraclass correlation (ICC) for T cells, cytotoxic T cells, helper T cells, and NK cells (ICC < = 0.05), and slightly higher ICC for B cells, dendritic cells, and monocytes (ICC = 0.16, 0.09, and 0.18, respectively) (Table 1). On the other hand, the hybrid approach had consistently low ICC for all seven cell subsets (0.005 to 0.055) (Table 1) demonstrating the improved reproducibility using the hybrid approach as compared to the manual approach over a broader range of cell subsets. Similar analyses on the eight subsets of cytotoxic and helper T-cells analyzed using the manual and k-means approaches showed very low ICCs for seven cell subsets (ICC < 0.03) using both methods while the ICC for the central memory CD4+ helper T-cells was slightly higher at 0.13 and 0.19 for the manual and k-means approaches respectively. (Table 2).

**Table 1:** Comparison of Inter and intra technician variability using hybrid and manual approaches for the seven major cell subsets evaluated in the PBMC controls.

Cell Types	Inter-variability		Intra-variability		Manual ICC	OpenCyto ICC
	Manual	OpenCyto	Manual	OpenCyto		
B cells	2.4E-05	2.54E-05	1.3E-04	4.32E-04	1.56E-01	5.54E-02
Helper T cells	2.48E-05	3.92E-05	1.54E-03	1.50E-03	1.59E-02	2.54E-02
Cytotoxic T cells	6.60E-27	7.01E-06	8.10E-04	1.32E-03	8.18E-24	5.29E-03
T cells	1.90E-18	2.92E-05	7.70E-04	9.91E-04	2.48E-15	2.87E-02
Monocytes	1.08E-04	1.78E-05	5.10E-04	6.39E-04	1.76E-01	2.71E-02
NK cells	1.20E-05	1.06E-05	2.30E-04	5.05E-04	5.01E-02	2.05E-02
Dendritic cells	3.83E-06	1.14E-05	3.96E-05	1.95E-04	8.82E-02	5.49E-02

**Table 2:** Comparison of Inter and intra technician variability using hybrid and manual approaches for the cytotoxic and helper T cell subsets evaluated in the PBMC controls.

Cell Types	Inter-variability		Intra-variability		Manual ICC	Kmeans ICC
	Manual	Kmeans	Manual	Kmeans		
Effector cytotoxic T cells	2.35E-04	7.50E-06	7.67E-03	2.06E-03	2.97E-02	3.63E-03
Effector memory cytotoxic T cells	2.68E-22	6.46E-05	8.16E-03	9.14E-04	3.28E-20	6.60E-02
Central memory cytotoxic T cells	9.83E-06	4.24E-05	2.40E-04	7.16E-04	3.94E-02	5.59E-02
Naïve cytotoxic T cells	2.36E-04	2.46E-04	2.32E-03	3.11E-03	9.21E-02	7.31E-02
Effector helper T cells	4.58E-06	8.42E-07	2.59E-04	4.62E-05	1.74E-02	1.79E-02
Effector memory helper T cells	1.82E-20	2.92E-23	2.13E-03	1.09E-03	8.54E-18	2.69E-20
Central memory helper T cells	3.54E-04	3.02E-04	2.25E-03	1.25E-03	1.36E-01	1.95E-01
Naïve helper T cells	1.98E-04	1.66E-04	2.35E-03	1.87E-03	7.78E-02	8.14E-02

### Time requirements

Based on the average time it took to manually analyze 931 samples, we estimated that the time requirement to manually analyze a single sample for 24 cell subsets was 7 minutes per FCS file. This translates to 109 hours for gating 931 samples and 1090 hours total for all 10,000 participants in the HRS immunophenotyping project. The average time to analyze all 24 cell subsets by automated analysis alone was 1.5 minutes/FCS file, which translated to 250 hours to analyze 10,000 files. However, since multiple OpenCyto jobs could be run in parallel, the overall processing time was <48 hours for the automated analysis. When determining time requirements for the hybrid approach the time for the automated process was added to the average time for manual guidance post pipeline per FCS file. For samples that required further manual refinement post automated pipeline, each FCS file took 1 minute to annotate in FlowAnnotator and 5 minutes to manually re-gate that respective sample in FlowJo (6 additional minutes after automated processing). Therefore, the time required for the hybrid approach when a file needed manual intervention was 7.5 minutes/FCS file which mirrored the manual gating approach time. However, Panel 1 had 29.1% (n = 2791) of its FCS files tagged for manual review requiring further refinement and Panel 2 had 23.7% (n = 2412) tagged leading to about 349 hours for the hybrid approach. Therefore, the automated (180 hours for 7209 samples) and hybrid (349 hours) approach together took 529 hours which is a 48% reduction in time as compared to a manual gating strategy. Individual populations that required more intensive manual refinements were the B lymphocyte subsets, while the T-helper and T-cytotoxic subsets were analyzed only by k-means clustering technique. Dendritic cells, NK cells, and monocyte subsets were processed through OpenCyto after minimal refinement and visualization in FlowAnnotator.

## Discussion

This study showed that a hybrid approach using hierarchical gating templates implemented in OpenCyto and a custom visualization tool, FlowAnnotator, can reproducibly and efficiently analyze multiparameter flow cytometry data and produce results comparable to manual gating strategies, thereby reducing the requirements for extensive technical experience that is necessary for robust and reliable manual gating strategies. This study provides a validation of a hybrid approach that incorporates automated gating strategies and manual refinement for use in large epidemiological studies.

The results of this study are consistent with previous studies that have evaluated the performance of other hybrid approaches for analysis for analysis of flow cytometry data. Hybrid analysis models such as FlowGM^5^ and DAFi^4^ that have incorporated automated and manual approaches have shown comparable results that are very similar to those observed in this study. The FlowGM model (now part of the OpenCyto pipeline) underwent a successful validation process similar to our study in a smaller sample size of 115 people^5^. The FlowGM model implemented pre-filtering gates for specific rare cell populations to improve the concordance in these smaller populations. In those comparisons, rare subsets of monocytes and NK cells had lower correlation with manual analysis, which is consistent with the results of our study. Thus, rare cell populations may need additional manual analysis to obtain accurate counts when utilizing hybrid approaches. Another comparable hybrid method, DAFi^4^ utilized manual gating strategies together with unsupervised clustering techniques (such as k-means and FlowSOM) and was validated against a manual gating strategy. The DAFi method showed that for a predefined cell population reproducibility using the hybrid approach was comparable to the manual approach in a small number of samples (n = 24). This is the first study to show comparability data with manual analysis for subsets of cytotoxic and helper T cells (naïve, effector, effector memory and central memory), as the previous hybrid studies did not present comparability data with manual analysis for these T cell subsets. Although both DAFi and FlowGM have shown that the choice of control samples has a minimal influence on final gating of immune cell subsets, this has not been systematically tested in large datasets. Since the HRS participants in this study represent a nationally representative sample of older adults over the age of 55 years that includes participants from both sexes, all racial groups and includes a substantial number of people with chronic diseases in addition to healthy individuals, the results of this validation is more likely to be generalizable to a broad population as compared to previous studies.

The FlowCAP study, which compared 36 automated and hybrid approaches with a gold standard set of manually gated samples determined that while fully automated algorithms aren’t suitable for all immune cell subsets they are excellent at delineating many different cell populations in diverse data sets^3^. FLOCK and flowDensity are 2 automated approaches that were the top performers when compared to manual gating in the FlowCAP assessement^3^. FLOCK utilized a grid-based partitioning and density distribution analysis to identify cell populations and can identify novel subsets that are not detected using standard two dimensional gating strategies^2^. However, the original FLOCK algorithm remains limited due to the small sample size that it was initially tested and created on (n = 8–17) indicating possible bias within the gating strategy and detection^2,12^. The flowDensity^10^ algorithm is based on a sequential bivariate gating approach that generates a set of predefined cell populations and is now incorporated into the OpenCyto pipeline. The findings from this study add to previous validation studies and confirms the feasibility of using a hybrid approach that utilizes both automated and manual strategies to accurately identify and quantitate cell populations. This study highlights the feasibility and advantages of hybrid approaches to achieve efficient and accurate immunophenotyping of all cell subsets in large population studies.

A strength of this study is the large amount of cell populations analyzed (24 cell populations) and the large sample size used for validation (n = 836 for Panel 1 and n = 757 for Panel 2). This study provides guidance on implementing quality control using sample population gates to accurately measure computational performance while optimizing measurement of individual cell populations. This study confirms previous findings that the output of automated high-dimensional gating methods will generally require manual post-processing to ensure valid results, especially for rarer immune subsets. Furthermore, since this analysis pipeline allows the output of workspace files compatible with commercially available software, this facilitates rapid post pipeline manual refinement for selected immune cell subsets.

Another strength is the use of a k-means clustering approach for identification of cytotoxic and helper T-cell subsets. The k-means clustering approach that simultaneously utilized 3 markers instead of 2 markers to identify subsets of cytotoxic and helper T-cells. This dramatically reduced variability in identifying these cell subsets when compared to a manual approach. In addition, these subsets analyzed by the k-means approach showed a reasonable correlation with results utilizing a standard manual approach, and this feasibility was further validated in FLOCK^2,14^. Thus, incorporating a k-means clustering approach may be beneficial in routine analysis of cell subsets that require >2 markers of robust identification. The strength of this analysis comes from the automated workflow used in conjunction with FlowAnnotator that decreases analysis time while still maintaining a supervised approach with the software. We optimized performance of hierarchical gating templates implemented in OpenCyto iteratively to increase the Pearson correlation value r > 0.80 prior to evaluating the remaining ~10000 HRS samples which still underwent a manual review in FlowAnnotator. This was facilitated by the use of CytoML^6^, which allows for the importing and exporting of gated cytometry data between commercial platforms (Cytobank, FlowJo, and Diva) and the R statistical programming language. FlowAnnotator facilitated combination of automated and manual data analysis and enabled successful large-scale implementation of a hybrid method in a large population study.

Utilizing the hybrid analysis model described here is an efficient alternative to the current gold standard of utilizing manual gating strategies as it greatly reduces processing time to analyze large datasets of multiparameter flow cytometry data. FlowAnnotator required 1–2 days to analyze 500 FCS files analyzed through the OpenCyto pipeline and batching the FCS files into groups of 500 allowed us to streamline the process. Hence, evaluating processing time requirements for individual validation projects prior to initiating large scale implementation will help optimize utility of FlowAnnotator. OpenCyto did not adequately identify rare cell populations (e.g. non-classical monocytes, the High CD56 subset of NK cells and the plasmacytoid dendritic cells). Thus, utilizing additional plug-in clustering algorithms (e.g.) k-means may be useful in reducing variability when analyzing rare cell populations and highlights the limitations of automated gating strategies in detecting rare cell subsets.

There are some limitations of our hybrid approach. Specifically, rare cell populations such as NK cell subsets and subsets of dendritic cells may still need to be analyzed manually until there are significant improvements in the algorithms to accurately identify rare cell susbets. Potential solutions to this problem may be to utilize large multi-dimensional gating strategies (e.g. k-means) to identify rare cell populations. We did show that simultaneously utilizing 3 markers to define cytotoxic and helper T-cell subsets can improve performance as compared to two-dimensional gating. This approach, in theory, be expanded to higher dimensional data as shown by other algorithms such as FLOCK that can use up to 19 dimensional data to identify rare cell populations^12^. However, trying to incorporate a fourth marker to better delineate subsets of effector memory and effector T cells was not successful in this study (data not shown) indicating that further work will need to be done to successfully utilize these multi-dimensional gating strategies to reliably define rare cell subsets of effector memory and effector T-cells and possibly other rare cell subsets as well.

Hybrid methods, such as those described in this study, have the potential to greatly speed up analysis of immunophenotyping data in large epidemiological studies and facilitate the implementation of FCM assays in population studies. Our validation process outlined here provides a framework for stepwise validation of a hybrid gating algorithm to manual gating. This study shows that automated analyses have not yet replaced manual gating for all cell populations and that manual gating will still be needed with automated algorithms for accurate estimation of rare or small cell populations. We have provided a framework for validation of a hybrid analysis method for use in epidemiological studies to facilitate efficient and accurate immunophenotyping analysis.

## Materials and Methods

### Study population

The Health and Retirement Study (HRS) is a longitudinal project sponsored by the National Institute on Aging and the Social Security Administration of American adults over 50 years old^17^. The biennial HRS interviews are conducted by trained survey interviewers employed by the Survey Research Center at the Institute for Social Research at the University of Michigan. At the 2016 HRS survey wave, venous blood samples were collected from 9,934 HRS participants in 7,227 households. Cryopreserved peripheral blood mononuclear cells (PBMCs) were obtained from a CPT^TM^ tube (BD Biosciences, San Jose, CA) as described previously^18^. The cryopreserved PBMCs were used for immunophenotyping in this study. All study methods were carried out in accordance with relevant guidelines and regulations^19^. This study was approved by the Institutional Review Boards at the University of Michigan, Ann Arbor and the University of Minnesota, Twin Cities. All HRS study participants provided a signed informed consent prior to study participation.

### Flow cytometry panels and detection of immune subsets

Two panels consisting of 17 fluorochrome-color cytometry panels targeting 24 cell populations were evaluated and have been described in detail previously^18^. The immune cell subsets were identified using minor modifications to the standardized protocol published by the Human Immunology Project^19^ to identify different subsets of effector cytotoxic T cells by including CD95 antibody. One vial of cryopreserved mononuclear cells containing ~4 million cells was thawed, and cells were incubated at 37 °C in RPMI media for 1 hour^20^. The cells were centrifuged at 1200 rpm for 10 min at room temperature. The cells were resuspended in 1X PBS and stained using the two antibody cocktails as outlined in Supplementary Table 3. The cells were kept on ice until analysis. All flow cytometry measurements were performed on a LSRII flow cytometer or a Fortessa X20 instrument (BD Biosciences, San Diego, CA). The percentage of cells in each cell subset was multiplied by the total white blood cell count to obtain absolute cell counts for all cell subsets.

### Manual data analysis

Using FlowJo (Tree Star Inc, v10), three analysts manually gated a total of 931 HRS study participants, all of which had at least 20,000 events. Initial filtering of data from each panel (a) delineated lymphoid or mononuclear populations using FSC-A/SSC-A profiles and (b) excluded doublets using FSC-A/FSC-H profiles and dead cells using FSC-A/fixable live-dead profiles. Subsequent gating identified major lymphocyte, dendritic cell, and monocyte cell populations. Guidance for gate placement was accomplished by optimizing peripheral blood mononuclear cells (PBMCs) controls using laboratory methods identical to those used in study samples. The overall gating strategy to identify the different cell types is shown in Supplementary Fig. 1.

### Automated data analysis

Automated data analysis was performed using OpenCyto, a Bioconductor framework that facilitates automated analysis of flow cytometry data by applying hierarchical gating strategies stored in text-based csv templates. We used hierarchical gating templates to delineate the step-by-step gating of an FCS file. Templates allow users to define the gating hierarchy (matching the process of manual gating) as well as adjust performance by selecting the algorithm used to generate a gate including built-in OpenCyto methods or plug-ins such as flowDensity. The templates also delineated the algorithmic parameters, such as the dimensions to be analyzed, whether a population is positive or negative for a given marker, the approximate range in the data where the gate should be placed, sensitivity thresholds for peak detection, number of expected populations, etc. We used CytoML’s GatingSet2flowJo API to export gates created in OpenCyto to a WSP file. WSP files were reviewed with FlowAnnotator and imported into FlowJo for further manual refinement if necessary.

### Post-processing visualization of data (FlowAnnotator)

Output WSP files from OpenCyto and paired text files with cluster assignment information were visualized using a custom software pipeline (FlowAnnotator), written in Java and developed by the Pankratz laboratory. FlowAnnotator automatically pre-generated an image file for each gate and displayed these images so that they can be quickly annotated. Pre-generating these images reduced the load time to a split second, allowing the analyst to review massive amounts of data in a short amount of time. In addition to reviewing all gates for one sample by pressing the up and down buttons, the same gate could also be viewed across different samples using the left and right buttons. This functionality is currently not available in commercial software and substantially sped up the annotation process when processing large numbers of samples. During this streamlined review process, the user annotated each image using a single keystroke with an annotation of its quality (e.g., “good”, “bad”, “needs manual refinement”, “low cell count”). We used these quality annotations to guide the iterative refinement of the hierarchical gating templates implemented in OpenCyto. Examples of acceptable and unacceptable gates are shown in Supplementary Fig. 2. Samples tagged for manual intervention were adjusted and exported as a WSP file through OpenCyto and manually gated in FlowJo (Fig. 3). Once the final gating was complete, the.fcs files and edited workspace files for the entire study was fed into the jFlow algorithm that computes the counts for each cell population defined in the workspace files. The pre-generated images were only 70 kilobytes in size on average, and so a large project of 10,000 samples with dozens of gates to be visualized would still easily fit on a 64 GB flash drive. The open-source repository for all three of these functions can be found here: https://github.com/PankratzLab/jFlow. FlowAnnotator is the graphical user interface portion of the jFlow package used to annotate the images.

### Optimization of hierarchical gating templates implemented in OpenCyto

After excluding files that were labeled with a “needs manual refinement” tag (n = 93 for panel 1 and n = 174 for panel 2) we utilized 836 FCS files in panel 1 and 757 FCS files in panel 2 for optimization of the OpenCyto template. Cellular populations chosen to serve as the optimization parameters were: T cells (CD3+/CD19−), B lymphocytes (CD3−/CD19+), dendritic cells (CD14−/HLA-DR+), NK cells (NK, CD16+/CD56+), and monocytes (CD20−/CD14+). If a hierarchical gate produced a correlation >0.75 between the OpenCyto template and manual gating for a subset, then the remaining samples underwent the same process for that subset. Populations reporting a Pearson correlation of r ≤ 0.75 were considered a failure of the automated analysis and were subjected to further iterative optimization using the hybrid method (OpenCyto+ manual review).

### Independent validation of the hybrid approach

Once the OpenCyto approach was optimized, the optimized protocol was compared to a manual analysis of the flow cytometry data in an independent dataset of 100 samples for all 24 cell subsets using Pearson correlation coefficients as described in the Statistical Analysis section.

### Differences in intra and inter-technician variability using hybrid and manual approaches

Eight internal peripheral blood mononuclear cells (PBMC) controls were analyzed twice a week concurrently with the HRS study samples for the seven major cell subsets (B lymphocytes, T cells, cytotoxic T cells, helper T cells, dendritic cells, NK cells, and monocytes) by five different technicians. These controls (n = 169) were run through the hybrid pipeline in conjunction with manual analysis in FlowJo, allowing us to compare inter/intra technician variability between our hybrid approach (OpenCyto+ FlowAnnotator) and manual approach (FlowJo). Since white blood cell (WBC) counts were not available for the PBMC controls, we expressed each cell subset as a percentage of the parent population and used the cell percentage to estimate technician variability. Linear mixed models were fitted to estimate the inter- and intra-technician variability and intraclass correlation (ICC), after controlling for the eight control samples (Tables 1 and 2). A low ICC would suggest little “technician” effect when measuring a specific cell subset. In addition to the analysis of the seven major cell subsets, we also used linear mixed models to estimate inter- and intra-technician variability and ICC among the cytotoxic and helper T-cell subsets identified using the k-means clustering approach.

### T cell subset k-means validation

Though most cell subsets are gated using two markers, the cytotoxic and helper T-cell subsets (effector, effector memory, central memory, and naïve) posed a unique challenge since they are better defined by three surface markers (CCR7, CD45RA, and CD28). While gating for these cell subsets using a manual method was performed mainly by using CCR7 and CD45RA with additional information from the CD28 marker being used as a secondary measure, the k-means clustering algorithm simultaneously utilized all three markers (CCR7, CD45RA and CD28) to accurately distinguish between these subsets of cytotoxic and helper T-cells. This required developing a method capable of gating in greater than two dimensions, which is not possible with standard two-dimensional gating techniques available in commercially available software such as FlowJo. To address this challenge, we used the k-means clustering algorithm (implemented in the “KMeans_rcpp” function of the ClusterR R package, version 1.1.0) to cluster the three-dimensional data into four subsets (using a pre-defined k of four). Input to the k-means algorithm comprised a sample’s cytotoxic and helper T cells with each of the three surface markers scaled to a standard deviation of one and mean of zero. Following k-means clustering, we assigned the four detected clusters as positive or negative for an individual marker based on the median marker value of all events in the cluster. For example, the two clusters with the highest median CCR7 value would be labelled CCR7+, and the remaining two clusters would be labelled CCR7−. We then assigned subset identity using the +/− labels of each cluster (e.g, the CCR7−/CD45R4+/CD28− would be labelled as the effector subset) using the k-means clustering method. Samples were flagged for manual gating when any cluster could not be mapped to a targeted subset. Lastly, the events in the four labelled subsets were assigned to their original parent (either CD8+ cytotoxic or CD4+ helper T cells) to obtain a total of eight populations. The labelled output from k-means was visualized and annotated using FlowAnnotator.

### Time requirements for manual analysis and OpenCyto

Manual time metrics were estimated by taking the average time spent on 1 FCS file from each analyst. Time metrics for running through the OpenCyto pipeline was based off running a 630 FCS file subset through the automated analysis pipeline. The time requirements for analyzing individual samples using both methods were then multiplied by the estimated sample size for the Health and Retirement Study (n = 10,000) to obtain the total analysis time by both methods for the entire study.

### Statistical analysis

We used percent bias, as a measure of the difference between the manual gold standard produced using FlowJo and the hybrid approach. If the difference was greater than zero, we determined there is positive bias in the automated analysis. The difference in mean values between the manual FlowJo counts and OpenCyto generated counts provided an estimate of the magnitude of difference between the methods. We also estimated Pearson correlation coefficients for all cell populations to determine the correlation between the two methods. We also performed sensitivity analysis by removing outliers (>3 standard deviations from the mean value) for all cell subsets and evaluated their influence on the observed correlation between hybrid and manual methods. We also estimated the F measure (the harmonic mean of precision and recall), due to its use from FlowCap study^3^, and its robustness to extreme outliers. Boolean gates were constructed to aid in calculating the F-score using the following formula *F* = *2TP/(2TP* + *FP* + *FN)*. Intra- and inter-technician variability were calculated using a linear mixed model with random technician effects, adjusting for the 8 PMBC controls as fixed effects. A conditional intraclass correlation was calculated as the proportion of total variance that occurs between technicians, i.e., inter-technician variance/(inter-technician variance + intra-technician variance). A low intraclass correlation would suggest little “batch” effects due to technicians. The analysis was performed for each of the seven major cell subsets using the hybrid approach (OpenCyto + FlowAnnotator) and manual approach (FlowJo) separately.

## Supplementary information

Supplementary Information.
