# Supplementary material for: Validation of a hybrid approach to standardize immunophenotyping analysis in large population studies: The Health and Retirement Study
Source: Sci Rep. 2020 May 29;10:8759. doi: 10.1038/s41598-020-65016-x (PMC7260195; doi:10.1038/s41598-020-65016-x)

**Supplementary Information**

Validation of a hybrid approach to standardize immunophenotyping analysis in large population studies: The Health and Retirement Study

DeVon Hunter-Schlichting^1^, John Lane^2^, Benjamin Cole^2^, Zachary Flaten^2^, Helene Barcelo^2^, Ramya Ramasubramanian^1^, Erin Cassidy^2^, Jessica Faul^3^, Eileen Crimmins^4^, Nathan Pankratz^2^, Bharat Thyagarajan^5,6^

^1^Division of Epidemiology and Community Health, University of Minnesota, Minneapolis, MN

^2^Divison of Computational Pathology, Department of Laboratory Medicine and Pathology, Minneapolis, MN

^3^Institute for Social Research, Survey Research Center, University of Michigan, Ann Arbor, MI

^4^Davis School of Gerontology, University of Southern California Davis, Los Angeles, CA

^5^Division of Molecular Pathology and Genomics, Department of Laboratory Medicine and Pathology, Minneapolis, MN

**Supplementary Table 1: Comparison of hybrid and manual approaches for all 24 cell subsets.**

| **Population** | **Population Mean^+^**  **(Mean Abs Difference)** | **F Score** | **n** | **Pearson Correlation after removing outliers** |
| --- | --- | --- | --- | --- |
| B lymphocytes | 0.166 (0.002) | 0.86 | 836 |  |
| T cells | 1.315 (0.064) | 0.95 | 836 |  |
| Cytotoxic T cells | 0.343 (0.013) | 0.92 | 836 |  |
| Helper T cells | 0.870 (0.057) | 0.96 | 836 |  |
| Monocytes | 0.253 (0.056) | 0.94 | 757 |  |
| Dendritic cells | 0.045 (0.0003) | 0.79 | 757 |  |
| Natural Killer | 0.236 (0.013) | 0.92 | 757 |  |
| B lymphocytes subsets |  |  |  |  |
| IgD+ memory | 0.014 (0.0003) | 0.75 | 836 |  |
| IgD- memory | 0.013 (0.002) | 0.73 | 836 |  |
| Naïve | 0.086 (0.003) | 0.83 | 836 |  |
| Cytotoxic T cell subsets |  |  |  |  |
| Effector | 0.17 (0.04) | 0.76 | 770 |  |
| Effector memory | 0.07 (0.03) | 0.64 | 770 |  |
| Central memory | 0.04 (0.02) | 0.55 | 770 |  |
| Naïve | 0.05 (0.03) | 0.72 | 770 |  |
| Helper T cells subsets |  |  |  |  |
| Effector | 0.02 (0.01) | 0.49 | 770 |  |
| Effector memory | 0.08 (0.06) | 0.62 | 770 |  |
| Central memory | 0.34 (0.05) | 0.84 | 770 |  |
| Naïve | 0.38 (0.07) | 0.92 | 770 |  |
| Monocyte subsets |  |  |  |  |
| Classical monocytes | 0.240 (0.050) | 0.75 | 757 |  |
| Non-classical monocytes | 0.009 (0.004) | 0.2 | 757 | 0.51 |
| Dendritic cell subsets |  |  |  |  |
| Myeloid dendritic cells | 0.032 (0.001) | 0.79 | 757 |  |
| Plasmacytoid dendritic cells | 0.007 (0.002) | 0.72 | 757 | 0.81 |
| NK subsets |  |  |  |  |
| CD56HI | 0.004 (0.005) | 0.35 | 757 |  |
| CD56LO | 0.218 (0.0005) | 0.91 | 757 |  |

^+^Absolute cell counts were used for these calculations and the mean absolute difference was calculated as the difference between manual and hybrid approaches. Thus, a positive value indicates that the manual approach counted more cells as compared to the hybrid approach.

**Supplementary Table 2. Validation of the final parameters utilized in the hybrid method with manual gating in an independent dataset of 100 samples.**

| **Population** | **Pearson Correlation^*^** |
| --- | --- |
|  | **(95% CI)** |
| B lymphocytes | 0.93 (0.92, 0.94) |
| T cells | 0.92 (0.92, 0.93) |
| Cytotoxic T cells | 0.90 (0.88, 0.92) |
| Helper T cells | 0.96 (0.96, 0.97) |
| Monocytes | 0.84 (0.82, 0.85) |
| Dendritic cells | 0.91 (0.88, 0.92) |
| Natural Killer | 0.93 (0.92, 0.93) |
| B lymphocytes subsets |  |
| IgD+ memory | 0.97 (0.96,0.98) |
| IgD- memory | 0.87 (0.85, 0.88) |
| Naïve | 0.92 (0.91, 0.92) |
| Cytotoxic T cell subsets |  |
| Effector | 0.83 (0.80, 0.86) |
| Effector memory | 0.61 (0.57, 0.65) |
| Central memory | 0.60 (0.55, 0.63) |
| Naïve | 0.79 (0.77, 0.83) |
| Helper T cells subsets |  |
| Effector | 0.75 (0.73, 0.77) |
| Effector memory | 0.77 (0.72, 0.80) |
| Central memory | 0.84 (0.83, 0.86) |
| Naïve | 0.89 (0.87, 0.90) |
| Monocyte subsets |  |
| Classical monocytes | 0.87 (0.86,0.88) |
| Non-classical monocytes | 0.59 (0.52, 0.64) |
| Dendritic cell subsets |  |
| Myeloid dendritic cells | 0.83 (0.80, 0.85) |
| Plasmacytoid dendritic cells | 0.82 (0.78, 0.83) |
| NK subsets |  |
| CD56HI | 0.44 (0.42, 0.45) |
| CD56LO | 0.92 (0.90, 0.93) |

^*^All Pearson correlations were statistically significant (p <0.05)

**Supplementary Table 3: Antibodies used in hybrid and manual approaches to identify 24 immune cell subsets**

| **CELL TYPE** | **MARKERS USED IN MANUAL APPROACH** | **MARKERS USED IN HYBRID APPROACH** | **PARENT POPULATIONS** |
| --- | --- | --- | --- |
| **B cell populations** |  |  |  |
| B lymphocytes | CD3- CD19+ | CD3- CD19+ | Single, live lymphocytes |
| IgD+ memory B cells | CD3- CD19+ IgD+ CD27+ | CD3- CD19+ IgD+ CD27+ | B cell |
| IgD- memory B cells | CD3- CD19+ IgD- CD27+ | CD3- CD19+ IgD- CD27+ | B cell |
| Naive B cells | CD3- CD19+ IgD+ CD27- | CD3- CD19+ IgD+ CD27- | B cell |
| **T cell populations** |  |  |  |
| T cells | CD3+ CD19- | CD3+ CD19- | Single, live lymphocytes |
| Cytotoxic T cells | CD3+ CD19- CD8+ CD4- | CD3+ CD19- CD8+ CD4- | T cells |
| Central memory cytotoxic T cells | CD3+ CD19- CD8+ CD4- CD45RA- CCR7+ CD28+ CD95+ | CD3+ CD19- CD8+ CD4- CD45RA- CCR7+ CD28+ | Cytotoxic T cells |
| Effector (E) cytotoxic T cells | CD3+ CD19- CD8+ CD4- CD45RA+ CCR7- | CD3+ CD19- CD8+ CD4- CD45RA+ CCR7- CD28- | Cytotoxic T cells |
| Effector memory (EM) cytotoxic T cells | CD3+ CD19- CD8+ CD4- CD45RA- CCR7- | CD3+ CD19- CD8+ CD4- CD45RA- CCR7- | Cytotoxic T lymphocytes |
| Naive cytotoxic T cells | CD3+ CD19- CD8+ CD4- CD45RA+ CCR7+ CD95- CD28+ | CD3+ CD19- CD8+ CD4- CD45RA+ CCR7+ CD28+ | Cytotoxic T cells |
| Helper T cells | CD3+ CD19- CD8- | CD3+ CD19- CD8- | T cells |
| Central memory helper T cells | CD3+ CD19 - CD8 - CD4+ CD45RA- CCR7- CD28+ CD95+ | CD3+ CD19 - CD8 - CD4+ CD45RA- CCR7- CD28+ | Helper T cells |
| Effector helper T cells | CD3+ CD19 - CD8 - CD4+ CD45RA+ CCR7- | CD3+ CD19 - CD8 - CD4+ CD45RA+ CCR7- CD28- | Helper T cells |
| Effector memory helper T cells | CD3+ CD19 - CD8 - CD4+ CD45RA - CCR7 - | CD3+ CD19 - CD8 - CD4+ CD45RA - CCR7- | Helper T cells |
| Naive helper T cells | CD3+ CD19 - CD8+ CD4 - CD45RA+ CCR7+ CD95 - CD28+ | CD3+ CD19 - CD8+ CD4 - CD45RA+ CCR7+ CD28+ | Helper T cells |
| **DC and NK populations** |  |  |  |
| Natural Killer lymphocytes | CD3 - CD19 - CD20 - CD14 - CD16+ CD56+ | CD3 - CD19 - CD20 - CD14 - CD16+ CD56+ | DC NK |
| Dendritic cells | CD3 - CD19 - CD20 - CD14 - HLA -DR+ | CD3 - CD19 - CD20 - CD14 - HLA -DR+ | DC NK |
| Myeloid dendritic cells | CD3 - CD19 - CD20 - CD14 - HLA -DR+ CD11c+ CD123- | CD3 - CD19 - CD20 - CD14 - HLA -DR+ CD11c+ CD123- | DC |
| Plasmacytoid dendritic cells | CD3 - CD19 - CD20 - CD14 - HLA -DR+ CD11c - CD123+ | CD3 - CD19 - CD20 - CD14 - HLA -DR+ CD11c - CD123+ | DC |
| CD56HI  Natural Killer lymphocytes | CD3 - CD19 - CD20 - CD14 - CD16+ CD56++ | CD3 - CD19 - CD20 - CD14 - CD16+ CD56++ | NK |
| CD56LO  Natural Killer lymphocytes | CD3 - CD19 - CD20 - CD14 - CD16+ CD56+ | CD3 - CD19 - CD20 - CD14 - CD16+ CD56+ | NK |
| **Monocytes populations** |  |  |  |
| Monocytes | CD3 - CD19 - CD20 - CD14+ | CD3 - CD19 - CD20 - CD14+ | DC NK MONO |
| Classical monocytes | CD3 - CD19 - CD20 - CD14+ CD16 - | CD3 - CD19 - CD20 - CD14+ CD16 - | MONO |
| Non-classical monocytes | CD3 - CD19 - CD20 - CD14+ CD16+ | CD3 - CD19 - CD20 - CD14+ CD16+ | MONO |

**Supplementary Figure 1a: Summary of gating strategy used for Panel 1 (lymphocyte panel)**


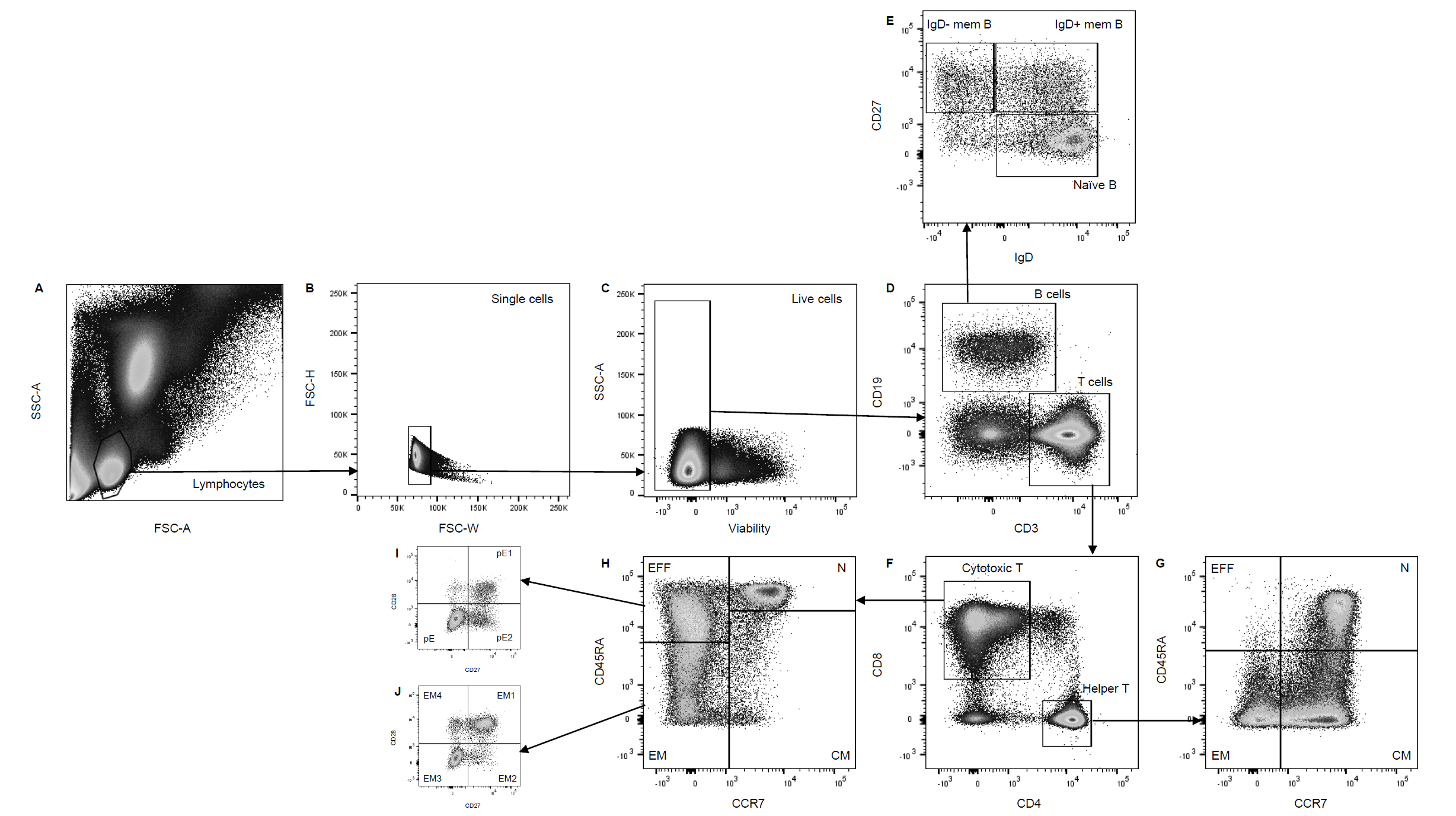


* The 24 immune cell subsets were measured using two flow cytometry panels. Supplementary Figures 1(a) and 1(b) show the gating strategies used to identify the various immune cell subsets in Panels 1 and 2 respectively.

**Supplementary Figure 1b: Summary of gating strategy used for Panel 2 (PBMC panel)**


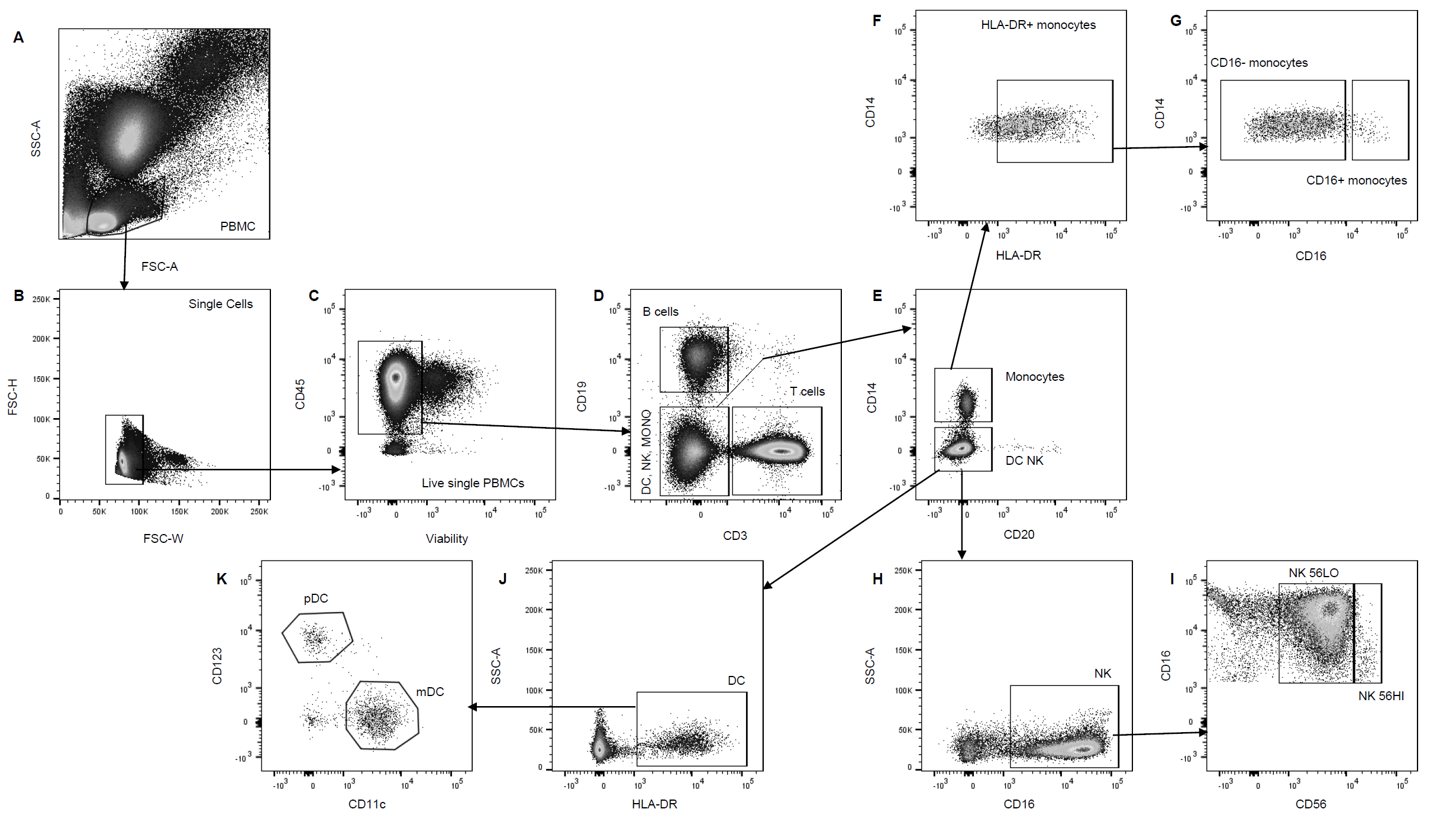


* Images from FlowAnnotator that demonstrate examples of acceptable gates that did not require manual refinement and unacceptable gates that required manual refinement.

**Supplementary Figure 2: FCS file images visualized in FlowAnnotator with examples of acceptable gates that did not require manual refinement and unacceptable gates that required further manual refinement using FlowJo.**

**Examples of gates that did not require manual refinement**

Helper T cells & Cytotoxic T cells

1. Markers for Helper T cells: CD4+ CD8-
2. Markers for Cytotoxic T cells: CD4- CD8+

Helper T cells (a) Cytotoxic T cells (b)


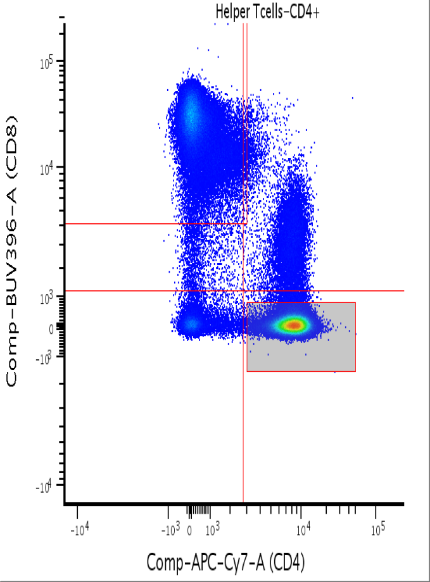

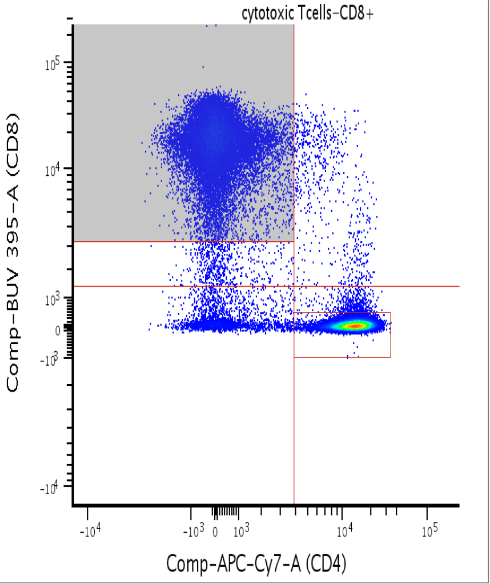


Example of good gate:

(a) The helper T cell gate is represented in the shaded region within the lower rectangle in the dot plot above. An acceptable helper T cell gate only includes the CD4+ CD8- population and does not include double positive population if present.

(b) The cytotoxic T cell gate is represented in the shaded region within the upper rectangle in the dot plot above. An acceptable cytotoxic T cell gate only includes the CD8+ CD4- population and includes all cells that variably express CD8+ as shown in the dot plot titled above.

Classical Monocytes

Markers: CD16- CD14+,


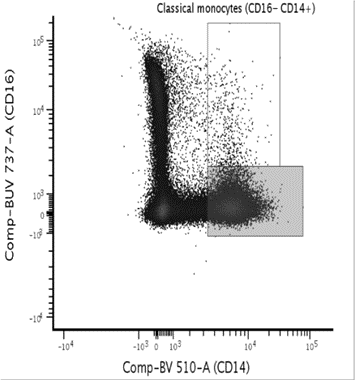


Example of good gate:

The classical monocyte gate is represented in the shaded region within the lower rectangle. An acceptable classical monocyte gate only includes the CD16+ CD14- population and doesn’t include any smearing of the dot plot (see above).

CD45+

Markers: CD45+


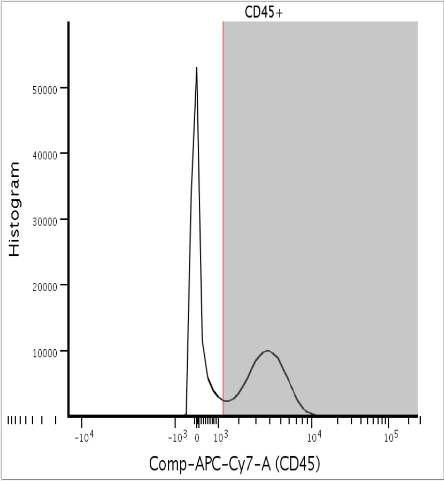


Example of a good gate:

The CD45+ population is represented as a histogram instead of a dot plot. A histogram was utilized for this population as the histogram was better suited to identify a single CD45+ peak, that is represented in the shaded region within the lower rectangle. A doublet peak, that would look like a 3^rd^ smaller peak after the second, would be included in the shaded portion.

## DC, NK, Mono

Markers: CD3- CD19-


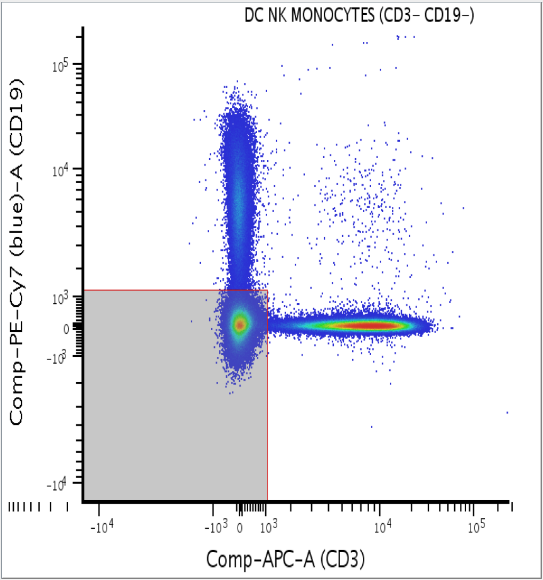


Example of a good gate:

This gate is represented in the lower left shaded rectangle. It only includes the CD3- CD19- population. T cell and B cell populations can be used to define cut offs for CD3 and CD19 axes respectfully.

Plasmacytoid DC Subset

Markers: CD11c- CD123+


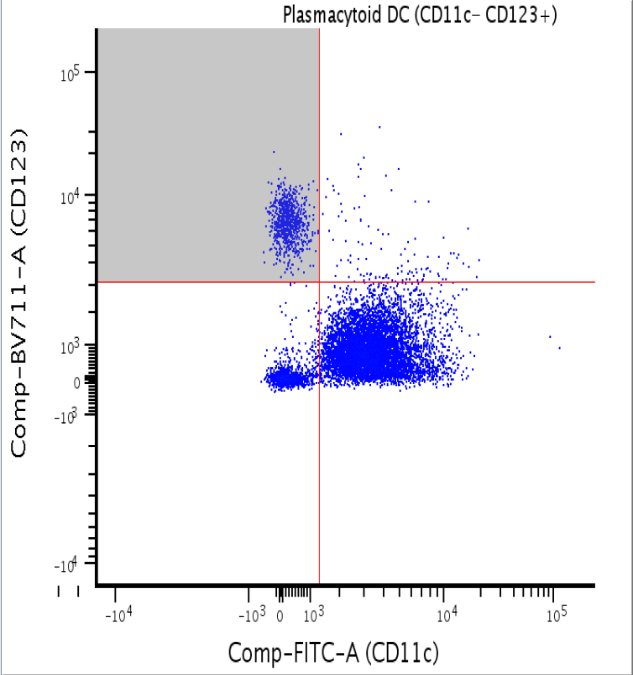


Example of a good gate:

The plasmacytoid dendritic cell subset is less frequent, represented in the upper left shaded rectangle and CD11c-/CD123+. The larger myeloid subset (CD11c+) is more frequent and often CD123- (seen in the unshaded lower right rectangle). However, neither gates should include the double negative population (seen in the unshaded lower left rectangle).

Live Single PBMC

Markers: SSC-A () FSC-A ()


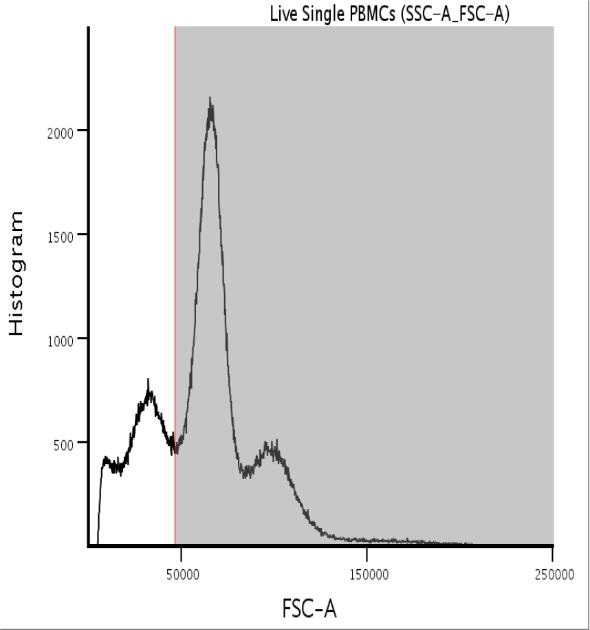


Example of a good gate:

This is another gate best visualized as a histogram. Here you can clearly see four individual peaks. Early validation studies using manual gating strategies confirmed that each peak represents a specific fraction. From left to right: **Peak 1** - cellular debris; **Peak 2** - red blood cells; **Peak 3** - lymphocytes and **Peak 4** - myeloid cells. It is a much clearer view than its dot plot counterpart, ideal for streamlining manual checking of post automated pipeline FCS files. An acceptable gate will not include the first two peaks and include all events in peaks 3 and 4 (lymphocyte and myeloid cells).

**Examples of gates that required further manual refinement**

**B cells**

Markers : CD19+ CD3-

*Manually tagged gate in FlowAnnotator*


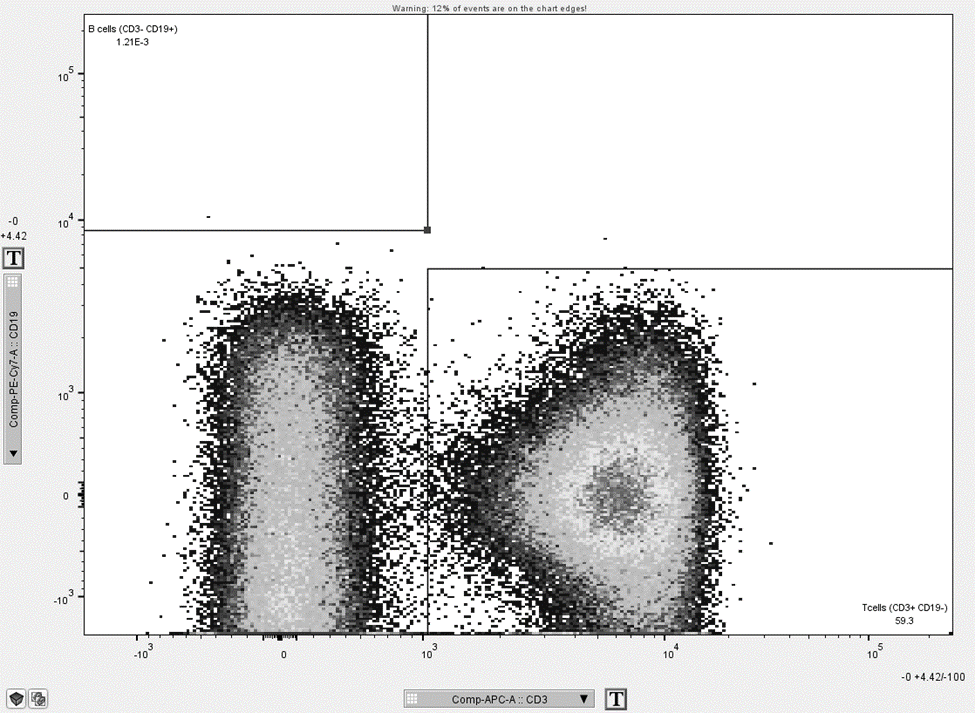


Reason for gate failure tag:

This is an example of a plot where ~12% of the “events” or cells were on the chart edge affecting the gate placement of the hierarchical gating template. This visualization above accounted for 88% of the manually failed FCS for the B cell population. The B cell gate is represented in the top left rectangle. Additionally, this sample was unique in addition to the axis shift of the dots, as the B cell population for this sample was rare.

This was corrected to include the missing events in the top dot plot visualization. The corrected image is visualized below.

*Manually accepted gate in FlowAnnotator after manual refinenment*


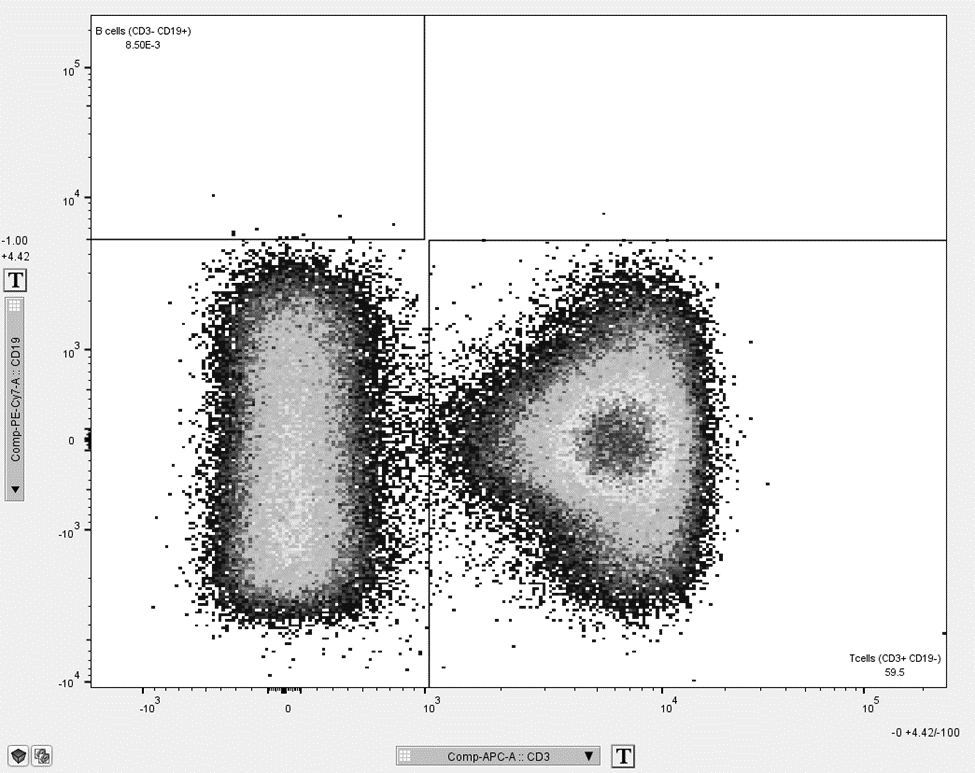


**B cell subsets (Inaccurate quantitation of IgD- memory B cells)**

Markers for Naïve B cells: CD27- IgD

Markers for IgD+ Memory cells: CD27+ IgD+

Markers for IgD – Memory cells: CD27+ IgD-

*Manually tagged gate in FlowAnnotator*


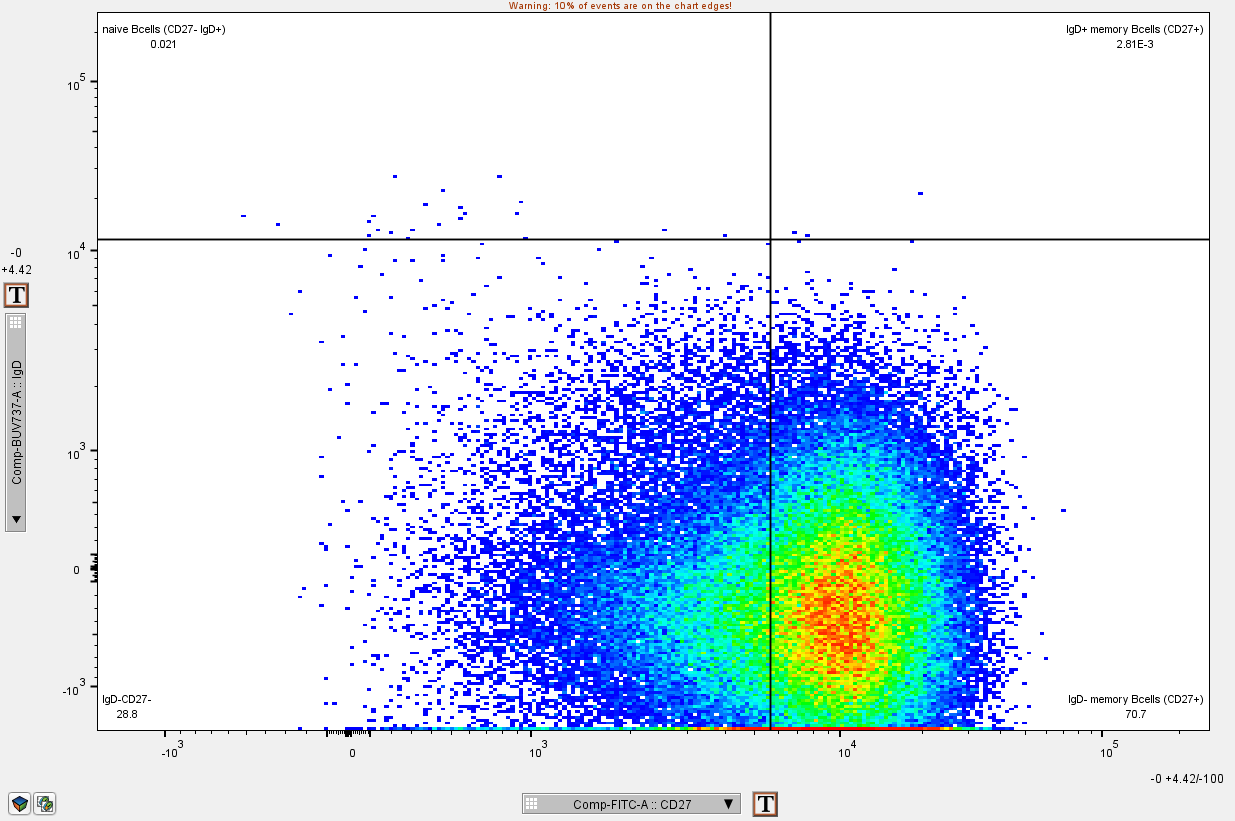


Reason for gate failure:

The CD27 axis is placed too far into the IgD+ memory B cell gate. This resulted in reduced numbers of IgD- memory B cells.

This was corrected by moving the CD27 gate to the left. This error accounted for 34% of manual tags in the B cell subset gates. The corrected image is visualized below.

*Manually accepted gate in FlowAnnotator after manual refinenment*


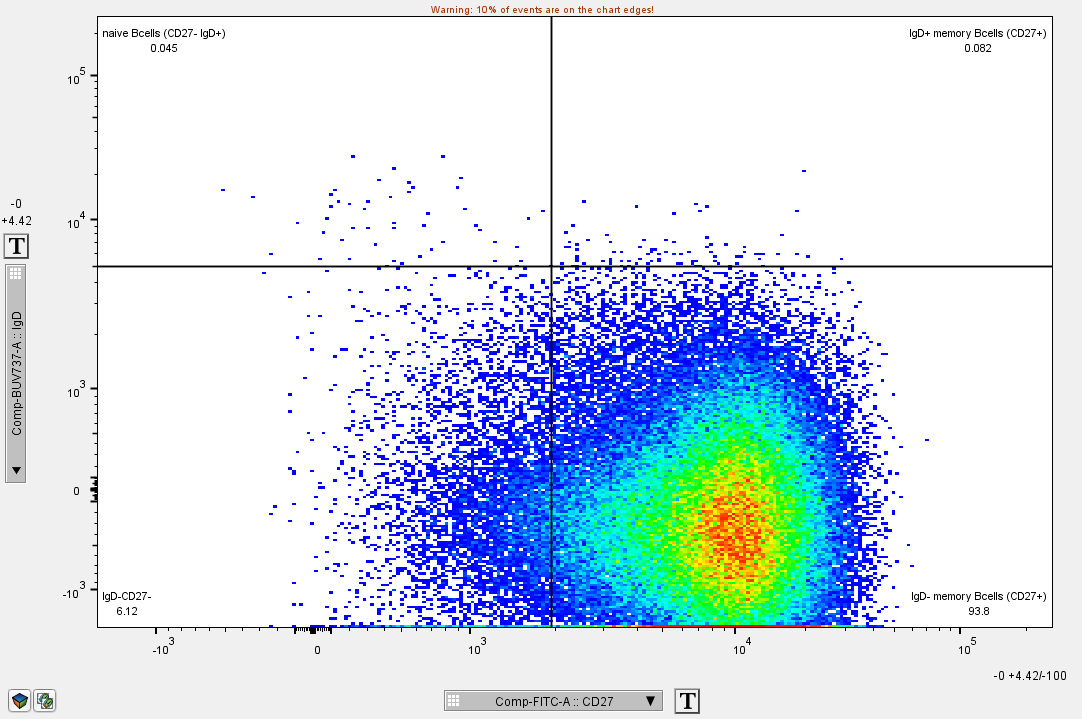


**DC Subsets (Inaccurate quantitation of myeloid DCs)**

Markers for Plasmacytoid DC: CD11c- CD123+

Markers for Myeloid DC: CD11c+ CD123-

*Manually tagged gate in FlowAnnotator*


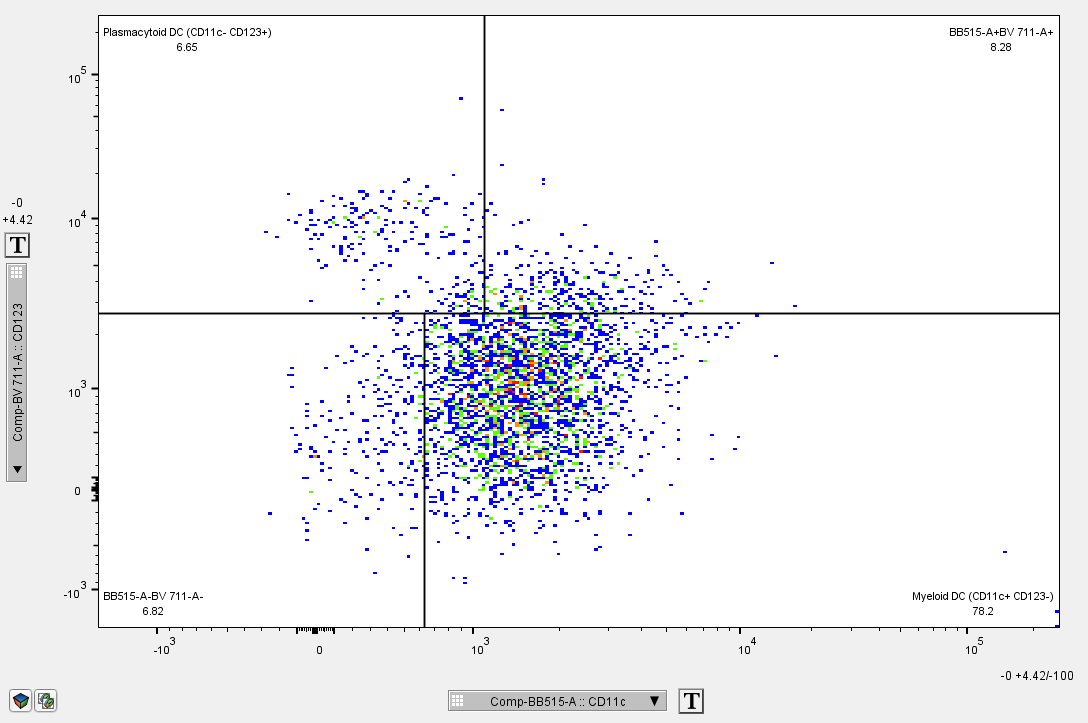


Reason for gate failure:

The cutoff for the CD123 axis excluded some myeloid DC cells and needed to be manually adjusted to get accurate counts of myeloid DCs.

This was corrected for by moving the CD123 axis to concurrently include more of the myeloid DC gate and refine the plasmacytoid gate. This accounted for 52% of the manual tags for the DC subset gates. This correction is visualized below.

*Manually accepted gate in FlowAnnotator after manual refinenment*


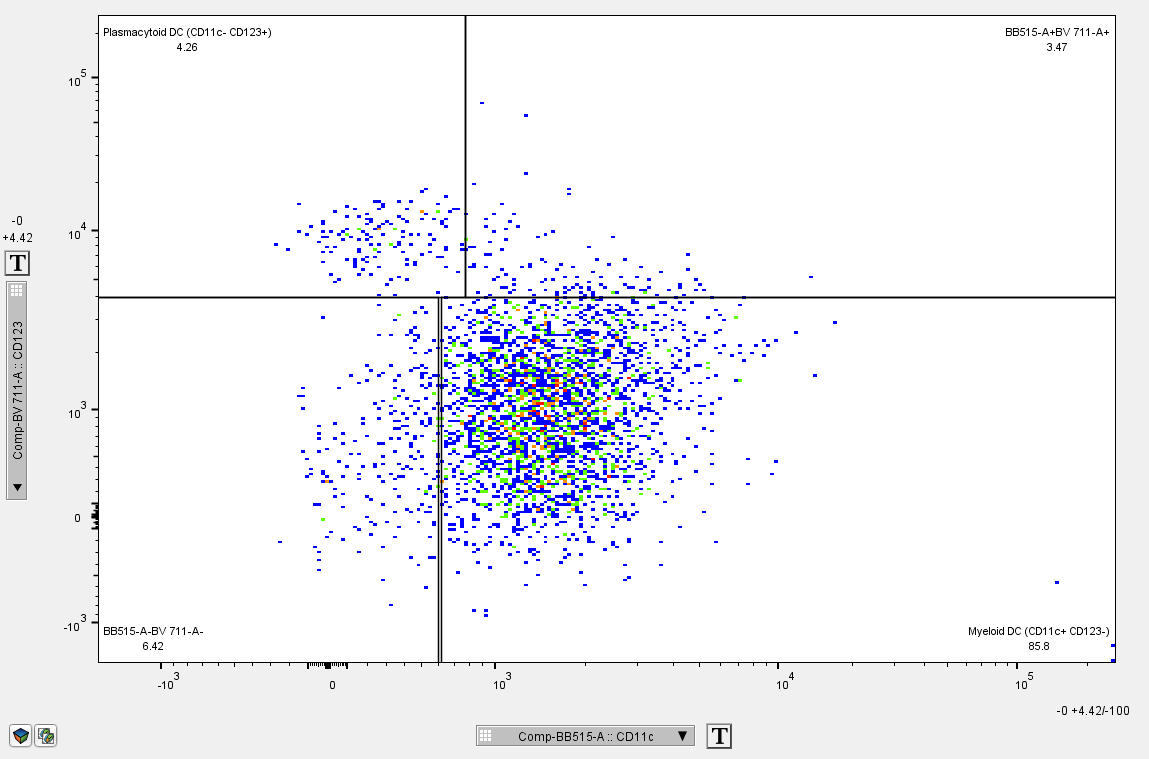


**kMEANS example (Inaccurate quantitation of central memory and effector memory cell subsets)**

Helper T (CD4+) subsets

Markers for Effecter Helper T cells: CCR7- CD45RA+

Markers for Effector Memory Helper T cells: CCR7- CD45RA+

Markers for Central Memory Helper T cells: CCR7+ CD45RA-

Markers for Naïve Helper T cells: CCR7+ CD45RA+

*Manually tagged gate in FlowAnnotator*


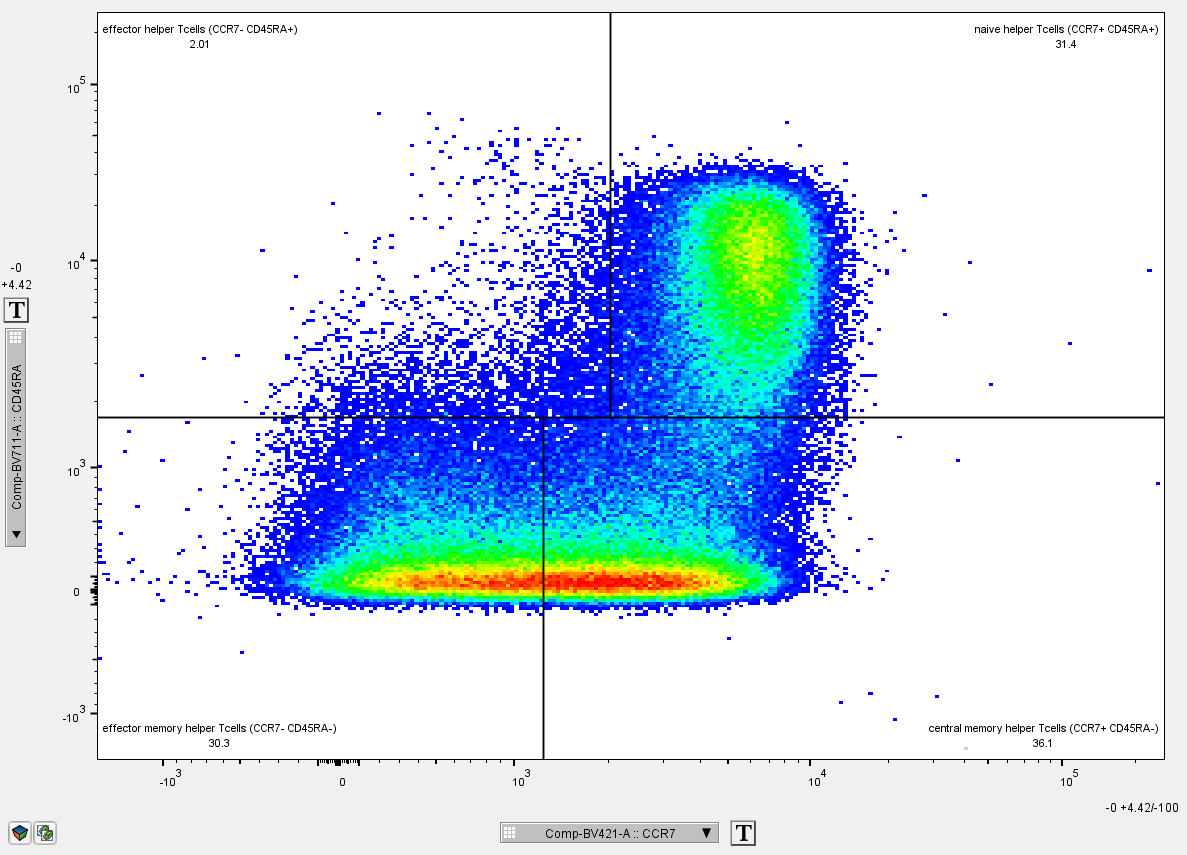


Reason for gate failure:

The CCR7 axis for the CD45RA- only gates is skewed too far to the right. This automated gating strategy resulted in a disproportionately large number of effector memory helper T cells and a reduced number of central memory helper T cells.

This was corrected for moving the CCR7 axis to the left. This error accounted for 42% of the manual tags in the helper T cell subsets. This correction is visualized below.

*Manually accepted gate in FlowAnnotator after manual refinenment*


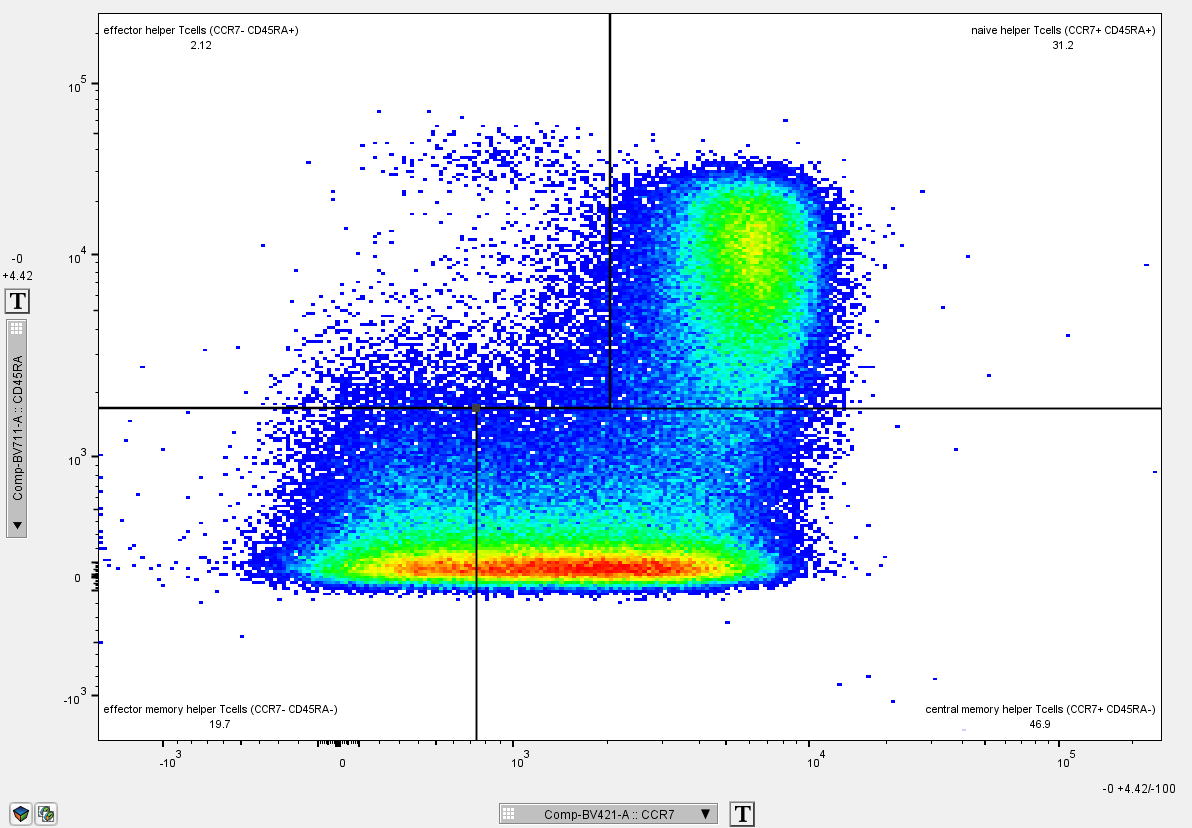

Supplement: Supplementary file 1 — Supplementary Information. [file 41598_2020_65016_MOESM1_ESM.docx]
